# Influence of Pitch Angle Errors in 3D Scene Reconstruction Based on U-V Disparity: A Sensitivity Study

**DOI:** 10.3390/s22010079

**Published:** 2021-12-23

**Authors:** Jonatán Felipe, Marta Sigut, Leopoldo Acosta

**Affiliations:** 1 Instituto Tecnológico y de Energías Renovables, 38600 Granadilla de Abona, Spain; 2 Departamento de Ingeniería Informática y de Sistemas, Facultad de Ciencias, Universidad de La Laguna, 38200 San Cristobal de La Laguna, Spain; marsigut@ull.edu.es (M.S.); lacosta@ull.edu.es (L.A.)

**Keywords:** sensitivity analysis, U-V disparity, 3D scenes reconstruction

## Abstract

U-V disparity is a technique that is commonly used to detect obstacles in 3D scenes, modeling them as a set of vertical planes. In this paper, the authors describe the general lines of a method based on this technique for fully reconstructing 3D scenes, and conduct an analytical study of its performance and sensitivity to errors in the pitch angle of the stereoscopic vision system. The equations of the planes calculated for a given error in this angle yield the deviation with respect to the ideal planes (with a zero error in the angle) for a large test set consisting of planes with different orientations, which is represented graphically to analyze the method’s qualitative and quantitative performance. The relationship between the deviation of the planes and the error in the pitch angle is observed to be linear. Two major conclusions are drawn from this study: first, that the deviation between the calculated and ideal planes is always less than or equal to the error considered in the pitch angle; and second, that even though in some cases the deviation of the plane is zero or very small, the probability that a plane of the scene deviates from the ideal by the greatest amount possible, which matches the error in the pitch angle, is very high.

## 1. Introduction

U-V disparity is a way of representing the information contained in a pair of stereo images to extract depth information. To do this, a three-dimensional space is built in the dimensions *u* (vertical coordinate of a point projected in one of the images of the stereo pair), *v* (horizontal coordinate of the same point), and Δ (difference between the horizontal coordinates of the point projected in both images of the stereo pair). This technique provides a satisfactory solution to the problem of obstacle detection, as has been shown in numerous works. For example, in [1,2] the V-disparity and U-V disparity are used, focusing on a specific subset of components of a scene for the detection and location of obstacles in vehicle guidance and driving aid systems. In [3], the authors propose a system that detects, recognizes, and tracks vehicles and pedestrians. They do this by combining different sources of local patterns and depth information using deep learning techniques. In particular, they employ an adaptive U-V disparity algorithm, the results of which are applied to a novel vehicle and pedestrian recognition system based on deep learning. In [4], we find another interesting contribution, in which an application is used to detect and predict the movement of pedestrians in various stages. First, the position of obstacles is precisely determined by merging the information provided by a stereo camera system and a laser scanner. The pedestrians are then identified using smart algorithms based on polylines and pattern recognition, as well as dense disparity maps and U-V disparity. In [5], the authors propose a procedure based on U-V disparity to detect and recognize obstacles on the road as part of a driver assistance system. The proposed realistic U-disparity map greatly improves accuracy when detecting distant obstacles compared to that obtained with a conventional U-disparity map. Other examples may be found in [6,7,8].

The 3D reconstruction problem, which consists of modeling a scene as a set of planes, can actually be regarded as a generalization of the obstacle detection problem, which is why it can also be successfully approached by making use of U-V disparity. For example, in [9], the authors propose a system for segmenting real-world scenes relying on a novel segmentation technique based on U-V disparity analysis to differentiate objects from the surfaces that support them in noisy environments. Both in [9] and in other similar papers, it is assumed that all the parameters that come into play are known exactly.

However, it is well known that in practice, discrepancies exist or may exist between the ’theoretical’ and ’actual’ values of certain parameters. These discrepancies can have different sources. They can be due to errors in determining the value of these parameters with complete accuracy, but may also be due to the changes that said parameters undergo over time due to the normal aging that is experienced by any physical system. Therefore, it seems reasonable to conclude that being able to determine how errors or uncertainties in the knowledge of certain system parameters affect the performance of an algorithm in real conditions is as important as having a procedure that satisfactorily solves the 3D reconstruction problem under ideal conditions. In other words, we wish to measure how sensitive the method designed is to the errors considered. The literature contains numerous works that support the importance of conducting a sensitivity analysis, as in [10,11,12,13]. Specifically, in [13], the authors state that sensitivity analysis is used to measure the robustness of model-based inference, that is, the extent to which the results output by the model depend on the assumptions used to construct it.

This type of analysis is also applied to systems in which depth information is obtained from a scene, which can be achieved using different techniques. For example, in [14], the authors propose what they call “The Relative Stereo 3D Measurement Method”. This method exhibits good tolerance to the calibration errors of the stereo vision system and is very precise in relative distance. In other applications, such as those found in [15,16], Kinect sensors are used to obtain depth information. In [15], the authors present a discussion on calibrating the Kinect sensor and discuss the accuracy and resolution of the depth information it provides. The experimental results obtained show that the random error in the depth measurements increases with the distance to the sensor. Elsewhere, [16] presents a mathematical model of uncertainty for the spatial measurement of visual characteristics using Kinect sensors. Thanks to this model, a qualitative and quantitative analysis is available for using Kinect sensors as 3D perception sensors. In [17], a different approach is presented in which the authors propose a method for analyzing and evaluating the uncertainties found in vision-based reconstructed scenes. This study considers primarily the errors in the image segmentation stage, which propagate throughout the reconstruction procedure.

In [18], we find a contribution that, despite its age, is of great interest. In this work, the authors conduct a thorough study of the influence of certain calibration parameters on the performance of a stereoscopic 3D reconstruction system. Specifically, they consider a binocular vision system with two cameras mounted on a rigid support and they study the depth errors due to the pitch, roll, and yaw between the two cameras. They also study the magnitude of the errors caused by the fact that in one of the two cameras the CCD array is not parallel to the lens, as well as the errors caused by lens distortion. Their quantitative study allows the authors to determine the requirements that the aforementioned parameters must meet for the errors to stay within certain thresholds. Finally, Ref. [19] provides a procedure for correcting misalignment in order to reduce depth errors due to camera displacements. The real images obtained with a stereo camera system show that the method proposed by the authors significantly reduces misalignments caused by the pitch, roll, and yaw of the camera.

The present article outlines an analytical study of how errors in the pitch angle of a stereoscopic vision system with respect to the world reference system influence the determination of the planes used to reconstruct a 3D scene. The pitch angle was chosen to carry out the study in line with the approach made by the authors of other works in which techniques similar to ours are used for modeling scenes, such as in [1,2]. As will be explained in Section 2, the influence of this parameter is quantified by measuring how these planes deviate in the presence of a certain error in the pitch angle with respect deviations under ideal conditions (assuming a zero error in the angle). At this point, it is worth asking whether a small error in the pitch angle can yield a large distortion in the reconstructed scene and if it is possible to limit the deviation suffered by the reconstructed planes as a consequence of the error in said angle. In this paper, we intend to answer these questions, something that, to the best of our knowledge, provides a novel contribution, since we have found no previous work that studies the sensitivity of the 3D reconstruction system to errors in the pitch angle of the vision system.

This work is structured as follows. Section 2 describes the vision system considered and the application of U-V disparity for modeling a scene. We also introduce an error in the pitch angle of the vision system to analyze how it influences the orientation of the planes that define the reconstructed scene. Section 3 describes the experiments carried out to measure the sensitivity of the system to errors in the pitch angle and the results obtained. Finally, Section 4 presents the main conclusions that we draw from the study.

## 2. Materials and Methods

Disparity is defined as the difference between the position of a pixel in the right image and its correspondence in the left image. Thus, a disparity map is understood as an image containing one disparity value for any point of the original image. By using a stereo system whose characteristics are known, it is possible to compute a full disparity map and calculate a complete 3D map from it.

### 2.1. Notation

**Figure 1:**
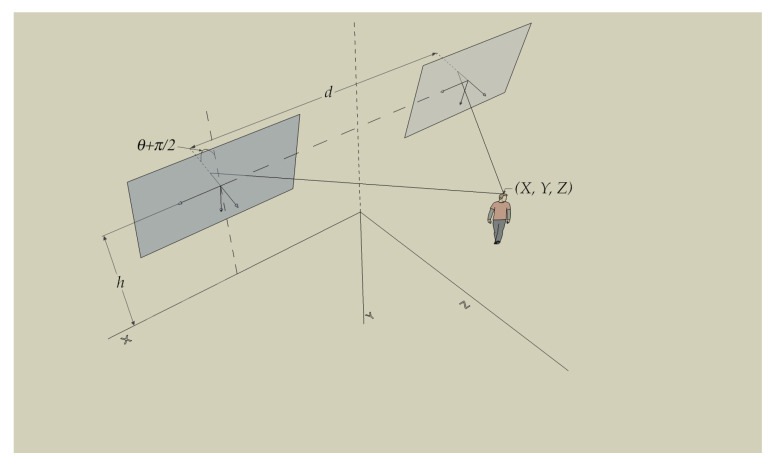
Pinhole vision system model.

A standard binocular vision system with a single degree of freedom (pitch angle) is shown in Figure 1.

The following parameters are defined:

- *d*: distance between the optical centers $\left(O_{i},i=r,l\right)$ of both cameras;
- *h*: height of the optical center of the camera above the ground;
- θ: the angle between the optical center and the horizontal plane (pitch angle).

In addition, three different reference systems are observed: $R_{w}$, which is the world coordinate system in which the real-world points $P={\left(X,Y,Z,1\right)}^{T}$ are represented, and $R_{l}$ and $R_{r}$, which define the coordinate systems of the left and right cameras. To completely define the framework, it is necessary to assume that the epipolar correction has been performed and that both image planes are coplanar and at the same level (h) above the ground.

In the $R_{l}$ and $R_{r}$ systems, each image point is designated by its coordinates (u,v), resulting from the projection of real-world coordinates in the image plane. A specific case is the projections of the optical centers, denoted as $\left(u_{0},v_{0}\right)$.

The intrinsic camera parameters required for the projection are denoted as:

- *f*: focal length of the cameras, assumed equal in both cameras;
- $t_{u},t_{v}$: size of the pixels in the *u* and *v* dimensions.

For simplicity, when expanding the equations $\alpha_{u}=f/t_{u}$ and $\alpha_{v}=f/t_{v}$ are used, assuming that $\alpha =\alpha_{u}=\alpha_{v}$.

The relationship between the world coordinate systems and the camera systems can be established by means of a simple transformation matrix.

### 2.2. Disparity Space

From the above equations, and using the epipolar constraint, we see that the coordinate v resulting from projecting point *P* is the same in both images, and has the form:

(1) $$\begin{array}{ccc} & & v=\frac{y}{z}=\frac{Y\left(\alpha \cos\theta +v_{0}\sin\theta \right)+\left(v_{0}\cos\theta -\alpha \sin\theta \right)Z^{\prime }}{Y\sin\theta +Z^{\prime }\cos\theta +h\sin\theta }\\ & & +\frac{\alpha h\cos\theta +v_{0}h\sin\theta }{Y\sin\theta +Z^{\prime }\cos\theta +h\sin\theta } \end{array}$$

Similarly, the $u_{i}$ coordinates for the left and right images are:

(2) $$\begin{array}{ccc} & & u_{i}=\frac{x_{i}}{z}=\frac{\alpha X+Yu_{0}\sin\theta +u_{0}Z^{\prime }\cos\theta -\alpha q_{i}\frac{d}{2}+u_{0}h\sin\theta }{Y\sin\theta +Z^{\prime }\cos\theta +h\sin\theta } \end{array}$$

Particularizing for the left and right images, $q_{i}=-1$ in the left image and $q_{i}=1$ in the right one, yields:

{(3a)ul=u0+αX+αd2Y+hsinθ+Z′cosθ(3b)ur=u0+αX−αd2Y+hsinθ+Z′cosθ

Hence, the disparity $\Delta =u_{l}-u_{r}$ is:

(4) $$\begin{array}{c} \Delta =\frac{\alpha d}{\left(Y+h\right)\sin\theta +Z^{\prime }\cos\theta }\\ \Delta =\frac{\alpha d}{\left(Y+h\right)\sin\theta +\left(Z+f\right)\cos\theta } \end{array}$$

As a result, the Disparity Space will be as defined in $\left(u_{r},v,\Delta \right)$.

### 2.3. Scene Model: U-V Disparity Generalization

Any scene in the real world can be interpreted as a combination of several planes with different orientations. With this assumption, every single object present in an arbitrary scene will be represented by a single plane whose orientation will be proportional to the position of the points in real-world space, defined by the general plane equation:

(5) rX+sY+tZ+u=0

In the state of the art, the implementations and developments based on V-disparity or U-V disparity are concentrated in a set of concrete planes that respond to reduced versions of Equation (5). For example, Ref. [1] presents the application of the V-disparity technique previously described in [20]. In this article, the authors reduce the recognized planes to those that respond to the equation tZ+u=0. In this way, they are able to estimate the inclination of the road and the existence of obstacles making use of only the information contained in the V-disparity plane.

On the other hand, in [2], not only the oblique planes are considered as in the previous case, but a distinction is also made in the vertical planes, which respond to Z=−u/t, and in those perpendicular to the plane image. In this case they make use of both the V-disparity and U-disparity projections to not only detect the elements of the image that respond to the cases considered, but also to determine their dimensions.

In this article, the authors do not limit the study carried out to a subset of planes that respond to some variation of the Equation (5), but rather propose a totally generalized treatment of the information. In this way, it is possible to carry out the composition of a complete scene and not only of certain elements that compose it.

#### Scene Elements Identification

It is necessary to identify which points belong to each element of the image in the U-V disparity space so that these points can be grouped and interpreted as a plane. Assuming this real-world simplification and taking into account the following theorem:

**Theorem** **1.** *Given two planes that are parallel to a line, the intersection of those planes will be another line parallel to the first.*

We can deduce that independently of the orientation of a given plane in the real world, is it always possible to find a line parallel to that plane that is also parallel to the image plane.

Introducing the restriction of Equation (5) and expanding from Equation (1), the disparity can also be calculated as:

(6) $$\begin{array}{cc} \Delta & =\frac{-2d}{2u+rd-2sh}\cdot \left(r\left(u_{r}-u_{0}\right)+\left(v-v_{0}\right)\left(s\cos\theta -t\sin\theta \right)+\alpha \left(s\sin\theta +t\cos\theta \right)\right) \end{array}$$

The Equation (6) can be particularized with the restrictions discussed above used by other authors, verifying that it is a generalization of those in [1,2].

Notice that the disparity is a function that depends on $u_{r}$ and *v*, which are discrete domain variables. Because of this, the gradient of the disparity is defined as the derivative of Δ with respect to both variables:

(7) $$\nabla \Delta =\left(\frac{d\Delta }{du_{d}},\frac{d\Delta }{dv}\right)$$

where:

(8) $$\frac{d\Delta }{du_{d}}=\frac{-2d}{2u+rd-2sh}r$$

(9) $$\frac{d\Delta }{dv}=\frac{-2d}{2u+rd-2sh}\left(s\cos\theta -t\sin\theta \right)$$

These equations make it possible to postulate a theorem that will be fundamental for this abstract representation of the content of a scenework:

**Theorem** **2.** *The gradient of every element $\left(u_{r},v\right)$ belonging to the projection and transformation of coplanar points in the real-world space of coordinates is constant and only depends on the plane itself and its orientation with respect to the vision system.*

**Figure 2:**
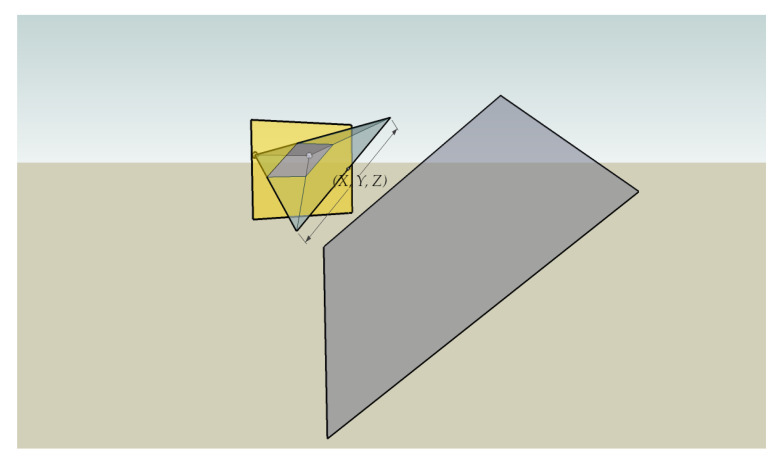
The arbitrarily oriented plane *P* (in gray) is represented in the image plane (in yellow). The spheres behind the image plane represent the optical centers of the left and right cameras, respectively.

An example of the application of Theorems 1 and 2 is shown in Figure 2. The colored parallel plane piped within the image plane is constructed by joining the projections of the line parallel to plane P in each image of the stereo pair. Since the line is parallel to the image plane, it will be contained in a plane described by the equation 0X+0Y+tZ=u and the angle between the planes will be 0. With this configuration and according to the previous expressions, we see that ∇Δ=(0,0), which means that the disparity is constant for every point on the line, with the two projections being parallel in the image plane.

Taking this into account, it is possible to divide the disparity space based on the different disparity gradients detected. We can thus obtain the equation of the plane that generates that gradient, making it possible to completely reconstruct the scene from the information in the $\left(u_{r},v,\Delta \right)$ space.

### 2.4. Scene Reconstruction

The above theorems allow us to lay the foundations for reconstructing any scene by representing it by means of a set of planes. Since our goal is to reconstruct a scene in world coordinates, we need to be able to calculate them based on the points projected in the vision system. Expanding the equations for $\Delta ,u_{r}$ y *v* (see Equations (1), (3b) and (4)), we find the expressions to obtain the coordinates of these points with respect to the world reference system:

(10) $$\begin{array}{cc} \;X & =\frac{d}{\Delta }\left(u_{r}-u_{0}\right)+\frac{d}{2} \end{array}$$

(11) $$\begin{array}{cc} Y & =\frac{d}{\Delta }\left(\left(v-v_{0}\right)\cos\theta +\alpha \sin\theta \right)-h \end{array}$$

(12) $$\begin{array}{cc} \;Z & =\frac{d}{\Delta }\left(\alpha \cos\theta -\left(v-v_{0}\right)\sin\theta \right) \end{array}$$

By using the property described in Theorem 2, we can recognize those projected points that belong to the same plane in the real scene. This makes it possible to have three pairs of points $\left(u_{ij},u_{dj}|j\in \left\{1:3\right\}\right)$ projected in both images that, once reconstructed, form part of the same plane. Moreover, we can calculate the disparity value $\Delta_{1}\cdots \Delta_{3}$ for each pair of points.

By specifying the Equations (10)–(12) for each pair of points, it is possible to calculate the coefficients of the equation of the plane. This can be done by simply determining those regions of space $\left(u_{r},v,\Delta \right)$ whose gradient is constant and selecting any three points within those regions.

From the expressions that represent the projected points on the image planes ((1), (3a), and (3b)) and the general equation of Plane (5), it is possible to analytically calculate the coefficients of the plane that contains the three points as described in Equations (13)–(16).

(13) $$\begin{array}{cc} \;r & =\frac{d^{2}}{\Delta_{1}\Delta_{2}\Delta_{3}}\alpha \left(\left(v_{2}-v_{1}\right)\Delta_{3}+\left(v_{1}-v_{3}\right)\Delta_{2}+\left(v_{3}-v_{2}\right)\Delta_{1}\right) \end{array}$$

(14) $$\begin{array}{cc} \;s & =\frac{d^{2}}{\Delta_{1}\Delta_{2}\Delta_{3}}\left(A\sin\theta +B\alpha \cos\theta \right) \end{array}$$

(15) $$\begin{array}{cc} \;t & =\frac{d^{2}}{\Delta_{1}\Delta_{2}\Delta_{3}}\left(B\alpha \sin\theta +Acos\theta \right) \end{array}$$

(16) $$\begin{array}{cc} \;u & =\frac{d^{2}}{\Delta_{1}\Delta_{2}\Delta_{3}}u_{1} \end{array}$$

$$\begin{array}{cc} u_{1} & =\left(\left(v_{2}-v_{1}\right)\frac{\Delta_{3}}{2}+\left(v_{1}-v_{3}\right)\frac{\Delta_{2}}{2}+\left(v_{3}-v_{2}\right)\frac{\Delta_{1}}{2}+\left(u_{d1}-u_{d2}\right)v_{3}+\left(u_{d3}-u_{d1}\right)v_{2}+\left(u_{d2}-u_{d3}\right)v_{1}\right)\alpha d\\ \; & +\left(B\alpha \cos\theta +A\sin\theta \right)h\\ A & =\left(\left(u_{d1}-u_{0}\right)v_{2}+\left(u_{0}-u_{d2}\right)v_{1}+\left(u_{d2}-u_{d1}\right)v_{0}\right)\Delta_{3}+\left(\left(u_{0}-u_{d1}\right)v_{3}+\left(u_{d3}-u_{0}\right)v_{1}+\left(u_{d1}-u_{d3}\right)v_{0}\right)\Delta_{2}\\ \; & +\left(\left(u_{d2}-u_{0}\right)v_{3}+\left((u_{0}-u_{d3}\right)v_{2}+\left(u_{d3}-u_{d2}\right)v_{0}\right)\Delta_{1}\\ B & =\left(u_{d2}-u_{d1}\right)\Delta_{3}+\left(u_{d1}-u_{d3}\right)\Delta_{2}+\left(u_{d3}-u_{d2}\right)\Delta_{1} \end{array}$$

**Figure 3:**
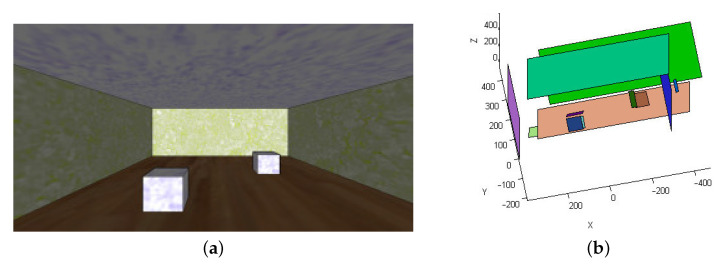
(**a**) Synthetic test scene; (**b**) reconstruction obtained from (**a**).

To illustrate the behavior of the method described in a simple way, Figure 3 shows its application on a synthetic image.

### 2.5. Pitch Angle Error Analysis

To introduce the effect of this error in the above expressions (13)–(16), the value of θ is replaced by θ+ε, where ε represents the difference between the calculated and ideal pitch angles. The ideal plane is obtained by assuming a zero error in the pitch angle.

#### 2.5.1. Measuring the Deviation between the Ideal and Calculated Planes

Given any three points $P_{1}-P_{3}$ belonging to the same element in the real scene identified by its coordinates with respect to the world reference system $\left[X_{j},Y_{j},Z_{j}\right]$, it is possible to determine the coefficients of the equation of the plane that contains them $P_{i,\mathrm{ideal}}={\left[r,s,t,u\right]}_{\mathrm{ideal}}$. If these points are projected using Equations (1), (3a), and (3b), it is possible to determine the projected coordinates in both images for all the points. Introducing the ε error factor described in the previous section and resorting to modified Equations (13)–(16) yields the coefficients of the reconstructed plane $P_{i,\mathrm{calculated}}={\left[r,s,t,u\right]}_{\mathrm{calculated}}$.

Since the planes are characterized by four parameters (r,s,t,u), one possible way to compare the ideal and calculated planes would be to obtain the difference between the values of these parameters in the two cases. However, it is easier and more intuitive to carry out this comparison using a single variable: specifically, the angle between the normal vectors of the two planes in question. This angle is calculated as follows:

(17) $$\varepsilon_{\mathrm{normal}}=\arccos\left(\frac{\left|{\vec{n}}_{\mathrm{ideal}}\cdot {\vec{n}}_{\mathrm{calculated}}\right|}{\left|{\vec{n}}_{\mathrm{ideal}}\right|\left|{\vec{n}}_{\mathrm{calculated}}\right|}\right)$$

The above equation makes it possible to quantitatively evaluate how the proposed reconstruction system responds to errors in the pitch angle.

#### 2.5.2. Construction of the Test Set

In order to conduct an exhaustive study of the behavior of the error in pitch angle and its influence on the reconstructed scene, we need to evaluate how the system responds in a variety of situations representing the cases that can occur in any a real scene. This problem is addressed by building a test set consisting of planes in a wide range of orientations with respect to the vision system. Note that from the point of view of the test set, it has no relevance that the planes that compose it come from a simple synthetic scene, such as the one shown in Figure 3, or from a more complex real scene.

The starting element for defining the set is a plane parallel to the image plane. This seed plane is rotated about the three Cartesian axes, varying the $\rho_{X}$, $\rho_{Y}$, and $\rho_{Z}$ angles between 0° and 90° at 5° intervals. The result is a test set consisting of 6859 planes with different orientations, but all at the same distance from the image plane. Therefore, in order for the test set to be able to faithfully represent any real scene, we must introduce an additional degree of freedom, namely, the distance from the planes to the image plane. This involves multiplying the size of the test set by a factor N, where N is the number of distances considered.

## 3. Results

To analyze how an error in the pitch angle affects the planes that are used to reconstruct a scene, we measured the difference between the normals of the ideal and calculated planes for this error for all the planes in the test set. Doing so revealed that the distance between the plane considered and the image plane has no influence on the deviation between the ideal and calculated planes. In other words, said deviation only depends on the orientation of the plane, and not on its distance from the image plane. This allows the test set to be reduced to 6859 planes with different orientations, as explained in Section 2.5.2, and at a distance of 5m from the image plane. The choice of this value for the distance is arbitrary.

**Figure 4:**
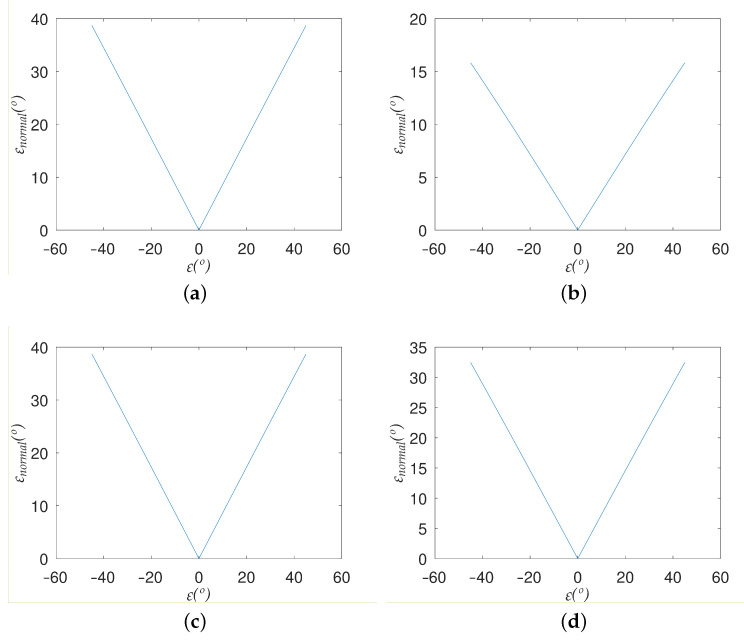
Deviation between the normals of the ideal and calculated planes as a function of the error in angle θ for: (**a**) Plane 1, (**b**) Plane 2, (**c**) Plane 3, (**d**) Plane 4.

Due to the large size of the test set, it is impossible to include in this paper the graphs showing the difference between the normals of the ideal and calculated planes as a function of the error in the pitch angle for all the planes in the test set. Since the qualitative behavior observed is the same for all of them, Figure 4 only shows the response for four planes with different orientations, which suffices to illustrate the diversity that exists at the quantitative level. The following planes were selected:

1. $\rho_{X}=0^{\circ };\rho_{Y}=45^{\circ };\rho_{Z}=45^{\circ }$
2. $\rho_{X}=15^{\circ };\rho_{Y}=75^{\circ };\rho_{Z}=0^{\circ }$
3. $\rho_{X}=30^{\circ };\rho_{Y}=15^{\circ };\rho_{Z}=90^{\circ }$
4. $\rho_{X}=45^{\circ };\rho_{Y}=75^{\circ };\rho_{Z}=0^{\circ }$

As Figure 4 shows, and, by extension, all the planes in the test set, there is a perfect symmetry in the deviation between the normals of the ideal and calculated planes with respect to ε. In other words, the value of the deviation only depends on the absolute value of the error in the angle θ and not on the sign. This allows us to characterize the effect of this error on the reconstruction of each plane by using a scalar parameter, which we will call deviationrate(dr), defined as:

(18) $$\mathrm{deviationrate}=\frac{\varepsilon_{\mathrm{normal}}}{\left|\varepsilon \right|}$$

This variable represents the slope of the line of the graph analogous to those shown in Figure 4 for that plane, leaving only the positive values of the error in the angle θ. This reduces the dimensionality of the problem and enables a compact representation on a single graph of the effect of ε on all the possible planes that are used to reconstruct a generic scene.

Despite this, we still have three independent variables that characterize every possible plane in the scene, $\rho_{X}$, $\rho_{Y}$, and $\rho_{Z}$, and a dependent variable, deviationrate. Thus, for the three-dimensional representation, we need to set the value of one of the independent variables, in this case $\rho_{X}$. The choice of fixed variable for the representation is arbitrary and does not adhere to any particular criterion.

**Figure 5:**
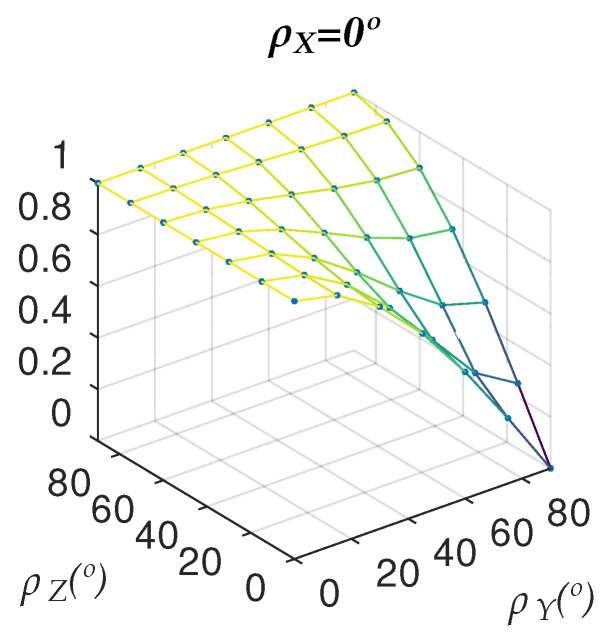
Deviationrate as a function of $\rho_{Y}$ and $\rho_{Z}$ and for $\rho_{X}=0^{\circ }$.

Figure 5 shows the graph corresponding to an angle $\rho_{X}=0^{\circ }$.

**Figure 6:**
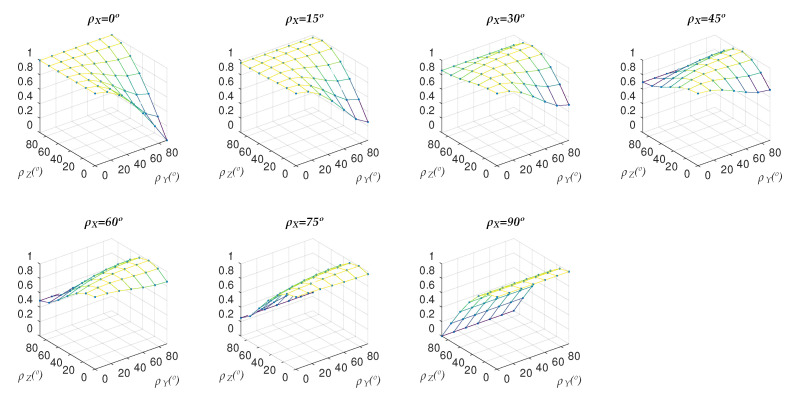
Deviationrate varying $\rho_{Y}$ and $\rho_{Z}$, for $\rho_{X}$ = 0°, 15°, 30°, 45°, 60°, 75°, 90°.

The observed behavior varies depending on the value of $\rho_{X}$ considered, as evidenced by Figure 6, which shows this same representation for values of $\rho_{X}$ between 0° and 90° with 15° increments between one graph and the next:

As Figure 6 shows, the value of deviationrate is bound between 0 and 1. Measuring this variable for all 6859 planes in the test set yields the same result in every case.

## 4. Discussion and Conclusions

The graphs shown in Figure 4 clearly demonstrate that the effect of an error in angle θ on the planes resulting from the reconstructed scene depends, in general, on the plane in question. Actually, as explained in the results section, it depends on its orientation with respect to the image plane, but not on its distance from it.

The results shown in Table 1 should be interpreted as follows: if we look, for example, at the row corresponding to the range 0.4 < dr <= 0.5, we see that 4.23% of the planes in the test set have a deviationrate in this range. Furthermore, 10.12% of the planes in the same set have a deviationrate less than or equal to 0.5.

In view of Figure 6 and Table 1, it holds that for all the planes in the test set, the value of deviationrate is always in the interval [0,1]. This implies that the angle formed by the normals of the ideal and calculated planes is, in the worst case, equal to the error in the angle θ.

This result is important, since it guarantees that the technique presented does not introduce errors in the reconstruction process, and limits the maximum error made during the process based on the deviation of the real vision system from the ideal.

**Figure 7:**
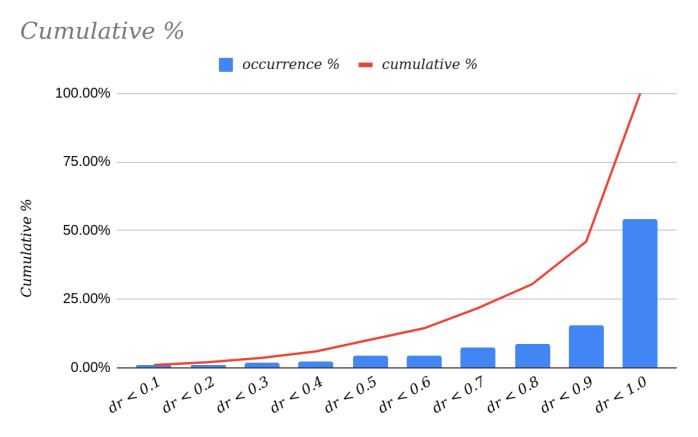
Graphical representation of the data in Table 1.

Analyzing the data further, the data contained in Table 1 are represented graphically in Figure 7. If we focus on the analysis of the cumulative percentage of possible values for the deviationrate variable, we see that it behaves like the density function of a random variable with a beta distribution; see [21,22], whose parameters are α=2 and β=0.4.

**Figure 8:**
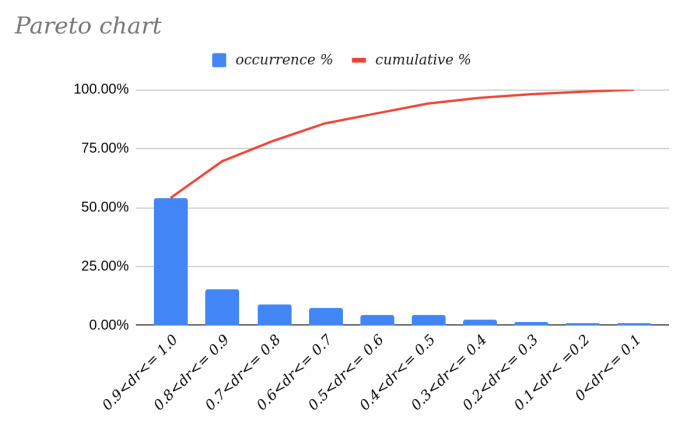
Pareto chart of the data in Table 1.

These results are supported by the Pareto chart [23] of the distribution in Figure 8. We can clearly see that, statistically, more than half of the planes will exhibit a deviation rate between 0.9 and 1, which means that the reconstruction error will be practically identical to that of the vision system used.

If we analyze the other end of the Pareto chart, we find a small percentage of planes whose deviationrate is 0 or practically zero. Focusing our analysis on these planes, we see that they correspond to those with $\rho_{X}=90^{\circ }$ and $\rho_{Z}=90^{\circ }$; that is to say, they are vertical planes perpendicular to the image plane and thus insensitive to errors in the pitch angle (θ).

The quantitative results presented earlier can be used to draw a series of conclusions to consider when using the scene reconstruction technique described. First, the sensitivity of the proposed system to errors in the pitch angle (θ) has been measured to reveal its upper bound. This allows us to guarantee that the plane obtained by the reconstruction will never deviate from the ideal by an angle greater than the error in the angle θ (ε).

In addition to the above result, it is possible to define a parameter that lets us anticipate the behavior of the reconstruction based on the error (ε) of the pitch angle. This parameter, the deviation rate, has been modeled as a random variable that follows a beta distribution. This result complements the previous conclusion since, in addition to having an upper limit on the error, the existence of a model allows us to determine what the expected deviation will be.

Analyzing the data in Table 2 obtained from the information shown in Table 1 and the Pareto chart in Figure 8, we may conclude that in 78.31% (8.69% + 15.56% + 54.06%) of the cases, the value of deviationrate will be greater than 0.7. This means that for almost 80% of the planes in the test set, the calculated plane will deviate from the ideal by an angle of at least 70% of the value of ε. In addition, in more than half of the cases (54.06%), the deviation will exceed this value by over 90%. It is also important to note that the results presented in this paper were obtained for a test set that contains 6859 planes with different orientations and at a fixed distance from the image plane, specifically 5m. The authors believe that the set of planes obtained in this way is sufficient to represent the diversity that can be found in a real scene, since, as has been proven, the deviationrate variable depends only on the orientation of the plane and not on its distance.

## Figures and Tables

**Table 1:** Left column: intervals of the deviation rate (between 0 and 1); center column: percentage of occurrence or percentage of planes in the test set that fall within each interval considered; right column: cumulative percentage or percentage of planes in the test set whose deviation rate is less than or equal to the upper value of the range considered.

Deviationrate	Occurrence %	Cumulative %
0.0 < dr <= 0.1	0.90%	0.90%
0.1 < dr <= 0.2	1.04%	1.94%
0.2 < dr <= 0.3	1.57%	3.51%
0.3 < dr <= 0.4	2.38%	5.89%
0.4 < dr <= 0.5	4.23%	10.12%
0.5 < dr <= 0.6	4.20%	14.32%
0.6 < dr <= 0.7	7.38%	21.69%
0.7 < dr <= 0.8	8.69%	30.38%
0.8 < dr <= 0.9	15.56%	45.94%
0.9 < dr <= 1.0	54.06%	100.00%

**Table 2:** Left column: intervals of the deviation rate (between 0 and 1); right column: percentage of occurrence or percentage of planes in the test set that fall within each interval considered.

Deviationrate	Occurrence %
0.7 < dr <= 0.8	8.69%
0.8 < dr <= 0.9	15.56%
0.9 < dr <= 1.0	54.06%
